# Innovative Approaches to Dental Education: Active Learning for Erosive Tooth Wear Diagnosis in Online and Face-to-Face Settings

**DOI:** 10.7759/cureus.98891

**Published:** 2025-12-10

**Authors:** Franciny Ionta, Daiana Martins, Gabriela Bueno, Wanderson Tosta, Amanda Ferreira, Eloiza Ferreira, Ana Paula Boteon, Mariana Braga, Daniela Rios

**Affiliations:** 1 Department of Pediatric Dentistry, Orthodontics, and Public Health, Bauru School of Dentistry, University of São Paulo, Bauru, BRA; 2 Department of Orthodontics and Pediatric Dentistry, Faculty of Dentistry, University of São Paulo, São Paulo, BRA

**Keywords:** active-passive learning, dental students, diagnosis, erosive tooth wear, online teaching

## Abstract

Objective

Active learning has been effective in improving diagnostic skills for dental caries, but its impact on erosive tooth wear (ETW) remains unclear. Additionally, the influence of online education in dentistry is not well understood. This study aimed to evaluate the impact of active learning, in both online and face-to-face (F2F) formats, on dentistry students' knowledge and diagnostic abilities related to ETW.

Method

Four groups of graduating dentistry students received different educational interventions: lecture online (L^on^) (n=8), active online (A^on^) (n=8), lecture face-to-face (L^F2F^) (n=14), and active face-to-face (A^F2F^) (n=14). The lecture covered theoretical knowledge of ETW, including etiology, diagnosis (including the Basic Erosive Wear Examination (BEWE) index), prevention, and treatment. Active learning involved exercises and discussions using clinical photographs for diagnosing ETW with the BEWE index. To assess the effectiveness of the educational intervention, students underwent two types of evaluation: a 12-item test to gauge their theoretical knowledge of ETW (applied pre- and post-intervention) and a 25-item quiz featuring intraoral photographs to evaluate their diagnosis performance (post-intervention). Additionally, the students' self-perception of the activity was assessed (post-intervention). Data analysis was performed by two-way analysis of variance (ANOVA) followed by the Tukey Test (p<0.05).

Results

In terms of theoretical knowledge of ETW, correct-answer rates increased for A^F2F ^(pre: 57.0% → post: 88.5%), L^F2F^(pre: 54.8% → post: 86.1%), and A^on^ (pre: 57.2% → post: 79.1%) in the post-intervention test, with no significant differences among them (p<0.05). L^on^ did not show significant improvement between pre (58.3%) and post (59.3%) tests (p>0.05). Regarding the clinical diagnosis of ETW, active learning groups (A^on^: 69.5% and A^F^^2^^F^: 64.7%) outperformed lecture-based approaches (L^on^: 47% and L^F2F^: 47,8%) (p<0.05). Importantly, there was no difference between online and F2Fsettings (p>0.05). Regarding self-perception, students participating in the online setting reported higher overall satisfaction and self-confidence compared with those in the F2F environment (p<0.001). However, no significant differences were observed between the groups when analyzing dissatisfaction levels (p=0.405).

Conclusion

Active learning enhances clinical diagnosis performance for ETW in both online and F2F settings and can improve theoretical understanding in the online context.

## Introduction

Traditional education in health professions often emphasizes theoretical learning, where students acquire knowledge in a classroom setting before applying it to patient care. However, practical training plays a crucial role in bridging the gap between theory and clinical practice. In dentistry, in particular, practical training is essential for developing a deeper understanding of diagnosis and treatment decision-making, as clinical practice requires not only theoretical knowledge but also the ability to apply it effectively in diverse scenarios. When students are not adequately prepared for clinical challenges, their ability to assess and manage cases effectively may be compromised, increasing the risk of diagnostic errors. Such errors are a major concern in healthcare, often leading to adverse events and malpractice claims [1].

One condition that exemplifies the challenges of clinical diagnosis in dentistry is erosive tooth wear (ETW), a pathological loss of dental hard tissue caused by intrinsic acids, such as gastric acid or extrinsic dietary acids, often exacerbated by attrition and abrasion [2]. Despite its high global prevalence (30%-50% in primary dentition and 20%-45% in permanent dentition), ETW is rarely diagnosed in its early stages, leading to underestimation and delayed diagnosis [3,4]. Therefore, gathering information from clinical signs, symptoms, and patient history is essential to establish a diagnosis and guide early treatment to prevent progression [5]. It is also crucial to ensure that students can accurately detect pathological tooth wear, which requires combining theoretical knowledge with practical experience [6,7]. Active learning methods have shown promise in diagnostic training, including dental caries [8]. Recent evidence indicates similar benefits for ETW, with students exposed to active learning showing greater diagnostic ability than those receiving traditional instruction [9].

Technological advancements have transformed education, enabling new teaching methodologies and reshaping how knowledge is acquired. The COVID-19 pandemic accelerated the expansion of online learning, influencing both current pedagogical approaches and future educational models [10]. The flexibility of online formats has facilitated remote access to academic content, including lectures, courses, and discussions via virtual platforms [11]. In health education, this transition required the reduction or restructuring of purely theoretical content and the incorporation of team-based, active, and self-directed learning strategies to address potential knowledge gaps associated with remote instruction [12-14]. This shift also extended to dental education, necessitating a comprehensive re-evaluation of several key aspects [14]. While dentistry cannot be entirely virtual, the future of dental education may incorporate a combination of face-to-face (F2F) and online components. To bridge this gap, the incorporation of active learning methods adapted for online environments can play an important role [14,15]. Despite the potential benefits, such as enhanced accessibility and a wider range of available content, the actual impact of these online learning methods on education remains to be assessed.

The primary objective of this study was to evaluate the effectiveness of online active learning in enhancing dental students' diagnostic skills for ETW, to assess whether the learning environment influences diagnostic accuracy. The formulated null hypotheses were H1: There are no differences between active learning and traditional learning regarding the diagnosis of ETW, and H2: There are no differences between online and F2F educational settings in the clinical diagnostic performance of ETW.

## Materials and methods

Ethical aspects

This study was approved by the local committee of research ethics (CAAE: 57811822.5.0000.5417), and all the participants had read and signed the informed consent form prior to participation. Due to ethical reasons, ensuring the benefits of an active learner-centered education approach, all the students underwent practical training following the final outcome assessment. The inclusion criteria were: third-year dental students enrolled in the Pediatric Dentistry course who voluntarily agreed to participate in the extracurricular activities. The exclusion criterion was incomplete participation in any stage of the intervention.

Study design

**Figure 1:**
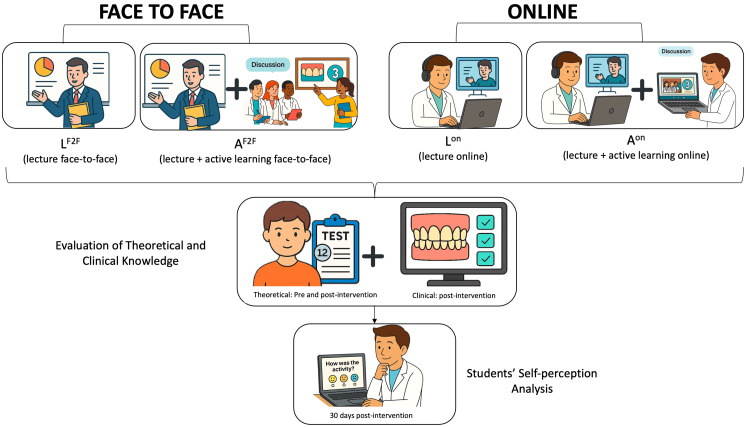
Study design of the educational intervention applied to dental students Schematic representation of the educational intervention applied to third-year dental students over two academic years. Participants were divided into four groups: L^F2F^, A^F2F^, L^On^, and A^On^. All students first completed a 12-item pre-test evaluating baseline knowledge on ETW. All groups attended a 60-minute lecture on ETW, but only the active learning groups (A^F2F^ and A^On^) participated in a subsequent 90-minute tutor-guided practical session using clinical images of extracted teeth. Post-intervention assessments included a theoretical test and a clinical diagnostic quiz based on new images. Finally, students’ self-perception regarding the activity was surveyed through a structured questionnaire. ETW: erosive tooth wear; L^F2F^: lecture face-to-face; A^F2F^: active face-to-face; L^On^: lecture online; A^On^: active online Image created by the authors with the support of artificial intelligence tools (ChatGPT/DALL·E, OpenAI).

The methodology of the present study was adapted from Braga et al. and Di Leone et al., and the study design is represented in Figure 1 [8,9]. The study was conducted in two distinct periods with a one-year interval. The sample size was estimated prior to data collection based on a two-way repeated measures analysis of variance (ANOVA) followed by Tukey’s post-hoc test. The calculation assumed a standard deviation of 10 and a minimum detectable difference of 17 units, based on values obtained from the study by Leone et al., 2024. The significance level (α) was set at 5%, and the type II error (β) at 20%, corresponding to a statistical power of 80%. Based on these parameters, the estimated sample size was approximately eight participants per group, totaling 32 participants overall. This study used a convenience sample, as all third-year dental students from the same cohort were invited to participate. The final sample size was determined by the number of students who voluntarily consented to take part, resulting in a total of 44 participants, exceeding the minimum required sample size and ensuring adequate statistical power for the analyses performed. The sample size calculation was performed using G*Power software (version 3.1, University of Düsseldorf, Germany). The study employed a single-blind design. Specifically, the data analyst was blinded to the group allocation throughout the statistical analysis, preventing potential bias in data interpretation. Full blinding of participants was not possible due to the nature of the educational intervention; however, the single-blind approach ensured objective analysis of the outcomes.

In the first year, F2F teaching activities were conducted following the methodology of Di Leoni et al., while in the following year, online teaching activities were carried out with a different group [9]. For the F2F group, 47 students were invited and 28 consented to participate. For the online group, 44 students were invited and 16 provided consent to participate in the study. Thus, randomization was performed only between the control group, which received a lecture as an intervention, and the test group, which received lectures with active learning. There were four groups under study: lecture online (L^on^) (control group online), active online (A^on^) (test group online), lecture face-to-face (L^F2F^)(control group F2F), and active face-to-face (A^F2F^) (test group F2F). The F2F lectures and activities were conducted at the university, where practical training involved the use of naturally extracted teeth, allowing for direct visual examination, observation of clinical photographs, and discussion with the instructors. In the online activity, clinical diagnoses were made on the same teeth, which exhibited different levels of ETW and had been photographed from all angles (mesial, distal, labial/buccal, lingual, and incisal/occlusal) for virtual use.

First, all participants (28 in the F2F group and 16 in the online group) answered a pre-intervention test consisting of 12 items about the definition, etiology, and general aspects of ETW, in order to assess baseline knowledge of the condition [9]. Then, all groups attended a 60-minute theoretical lecture with a professor who has expertise in ETW. Following this, only the A^on^ and A^F2F^ participants underwent a 90-minute theoretical-practical tutor-guided session, discussing a collection of 25 intra-oral or extracted teeth’s photographs.

At the end of the groups’ intervention, the participants of all groups completed the 12-item online test on their general knowledge of ETW again. Additionally, their clinical diagnosis performance of ETW was evaluated by a quiz, a 25-item set of intraoral photographs depicting deciduous and permanent teeth. The Basic Erosive Wear Examination (BEWE) scores were used to evaluate the degree of tooth wear [16]. It should be noted that the photographs utilized for evaluation were different from those presented during the practical training session. At the end of the activity, students' self-perception was assessed using a questionnaire.

Training of tutors

The team of tutors consisted of postgraduate students (master’s and doctoral students). Their training and calibration were designed to ensure they completed all stages of the activity proposed for the undergraduate students. Initially, their residual knowledge about ETW was assessed through a questionnaire, and their diagnostic skills were evaluated using an image-based exercise with photographs and extracted teeth. Following this, they attended a 60-minute theoretical class on the subject, after which they completed the same questionnaire and image-based exercise again. Finally, they received practical training on diagnosing ETW using the BEWE scoring system, and the theoretical questionnaire was administered once more. The results of the questionnaires and the correct answers from the image-based exercise at three different points in time (baseline, post-theoretical class, and post-practical training) were compared. To be selected as a tutor, postgraduate students were required to achieve at least 80% accuracy in the assessments, ensuring they were qualified and calibrated to assist with the activity for the undergraduate students. After one year, the same tutors were trained again.

All the nuances of implementing the activity, as well as the logistics required for conducting the study, were identified and adapted for online teaching, following the parameters of the F2F activity [9].

Lecture online (L^on^) and face-to-face (L^F2F^) groups

The students were assessed on their theoretical knowledge through a questionnaire containing 12 questions about ETW. They then attended a 60-minute online (L^on^) or face-to-face (L^F2F^) theoretical class based on the conventional teaching model, delivered by an expert in the field (DR). The class focused on ETW and its clinical diagnosis. Immediately after the class, students completed an online questionnaire that presented 25 photographs of primary and permanent human teeth, with and without various levels of erosive wear, to perform a "clinical" diagnosis using the BEWE index scores. Before leaving the activity, they answered the same 12-question theoretical knowledge questionnaire again. In a subsequent step, after this immediate assessment, participants in this group were offered theoretical-practical training to ensure all attendees received comparable learning. However, only the results from the assessments prior to the practical training were included in the data analysis for this group.

Lecture and active learning online (A^on^) and (A^F2F^) groups

 In this group, students first completed the questionnaire with 12 questions assessing their theoretical knowledge of ETW. They then attended a conventional theoretical class alongside the online control groups. Following the class, they were divided into pairs and participated in a complementary online or F2F activity, which consisted of a 90-minute theoretical-practical training session facilitated by tutors/monitors. This session involved discussions and exercises using 25 pre-selected clinical photographic images and extracted teeth illustrating various levels of ETW in the primary and permanent dentitions. In the F2F activity, students evaluated clinical images and extracted teeth, while in the online activity, the same extracted teeth were photographed from five different angles for evaluation. At the end of the training, the test group completed a practical evaluation, analysing 25 teeth to diagnose ETW using the BEWE index, identical to the assessment given to the control group. Before concluding the activity, they repeated the questionnaire on their theoretical knowledge of ETW.

Basic Erosive Wear Examination (BEWE) scores

The analysis of ETW was performed using the BEWE classification. The BEWE was developed as a simple scoring system to record clinical findings and assist in the diagnosis and management of ETW. A score of 0 is given for teeth with no enamel surface loss, while a score of 1 is assigned for initial loss of enamel texture. If there is hard tissue loss involving dentin but affecting less than 50% of the surface, the score is 2. A score of 3 is assigned when more than 50% of the surface is affected [16].

Activity dynamics and outcome assessment

The F2F teaching activities have been previously described in detail [9]. The students who agreed to participate in the online phase attended the university in person to minimize external interference during the activity. They used their own laptops with headphones individually. After the 60-minute online theoretical class, the groups were divided and relocated to two separate rooms so that the online training and assessment could take place simultaneously.

During the online training, students evaluated each designated surface on the images of the selected teeth. If ETW was detected, they assessed its severity using the BEWE scores. The tutors provided feedback on the exercises and addressed any questions raised by the online pairs. In cases of disagreement between the participants in the pair, the images were re-evaluated with the assistance of the tutors to ensure a deeper understanding of the subject and clarify any doubts or errors. A set of 25 clinical images, different from those used in the assessment, was employed for the training. Additionally, 10 images with five different angles of extracted human teeth, either primary or permanent, with or without ETW, were used.

After the practical training, the A^on^ group completed the same assessment taken by the L^on^ group, allowing for performance comparison between the two groups. To evaluate the effectiveness of active teaching-learning methods, theoretical knowledge was assessed before and after the theoretical class using a 12-question multiple-choice test previously adopted in Di Leone et al. [9]. This questionnaire was developed based on content from the ETW Foundation website to encompass both the knowledge domain and the students’ ability to contextualize situations involving the diagnosis of ETW (see Appendix) [17].

The practical assessment consisted of projecting 25 photographed teeth, with all surfaces shown. The students had to classify each tooth according to the BEWE score. They were given one minute to evaluate each image and determine whether or not ETW was present. If present, they classified the wear according to the BEWE score.

Students self-perception

Students’ self-perception (satisfaction, dissatisfaction, and self-confidence) regarding the activities was also assessed using a questionnaire with five questions, in which responses were rated on a scale adapted from Signori et al.: 1- slightly, 2- moderately, and 3- extremely [18]. The aim was to assess which of the two different educational approaches (interactive lectures online and active learner-centered education) and settings (online and F2F) led to greater satisfaction, better self-confidence, and lower dissatisfaction with the activities used to diagnose ETW. For statistical analysis, composite scores were calculated for satisfaction and self-confidence (sum of four positive questions regarding satisfaction with the activities, self-confidence in diagnosis, and theoretical knowledge) and dissatisfaction (sum of two negative questions regarding feelings of distress and tension about the activities). In this data processing, all response values (1, 2, or 3) were summed to transform the ordinal qualitative variable into a continuous quantitative variable, allowing for statistical analysis across all criteria. For positive questions, higher summed values indicated greater satisfaction, while for negative questions, higher summed values reflected higher levels of dissatisfaction.

Statistical analysis

After confirming the assumption of normal distribution using the Shapiro-Wilk test, a two-way ANOVA was performed, followed by the Tukey test, to compare: (1) the percentage of correct answers on the quiz of ETW’s clinical diagnosis applied post-group intervention, and (2) the students' self-perception post-group intervention (satisfaction, self-confidence, and dissatisfaction).

Two-way repeated measures ANOVA and Tukey’s Test were conducted to evaluate the percentage of correct answers on the 12-item test of theoretical knowledge of ETW applied pre-and post-group intervention. To assess the magnitude of differences in diagnostic performance, effect sizes (Cohen’s d) were calculated. For all analyses, a significance level of 5% was adopted. The statistical analysis was performed using the jamovi project (2024).

## Results

Sixteen participants for online strategies (L^on^=8; A^on^=8) and 28 participants for F2F (L^F2F^=12; A^F2F^=16) signed the consent form and carried out all the study activities, including the completion of pre-/post-interventions and self-perception questionnaires. Regarding ETW knowledge evaluation, the results showed interaction among the factors under study: time of application (pre- and post-intervention); learning strategy (interactive lecture and active learning methods); and setting (online and F2F). The percentage of correct answers on questionnaires of the evaluated groups is shown in Table 1.

**Table 1:** Mean values (±SD) of the percentage of correct answers on the theoretical knowledge evaluation of ETW (pre- and post-intervention 12-item test) for online and F2F settings ^a, b^Statistically significant differences. Moment (pre-and post-test): p<0.001; moment and group (L and A): p=0.022; moment and setting (On and F2F): p<0.001. Significant interaction (moment, group, and setting): p=0.024 (two-way repeated measures ANOVA and Tukey’s Test). ANOVA: analysis of variance; ETW: erosive tooth wear; F2F: face-to-face

Group	^On ^(Online)			^F2F ^(Face-to-face)		
	n	pre-test	post-test	n	pre-test	post-test
L (lecture)	8	58.3 (±10.9)^a^	59.3 (±15.7)^a^	12	54.8 (±7.4)^a^	86.1 (±8.0)^b^
A (lecture+active learning)	8	57.2 (±10.4)^a^	79.1 (±14.1)^b^	16	57.0 (±12.5)^a^	88.5 (±5.2)^b^

Only L^on^ did not show significant improvement in the percentage of correct answers post-intervention. A higher percentage of correct answers was found on the post-intervention questionnaire for A^F2F^, L^F2F^, and A^on^, without significant differences among them (p<0.001). The percentage of correct answers regarding basal knowledge was similar between the two learning strategies and settings.

The results of the ETW diagnosis ability of the students (Table 2) showed that all groups exposed to active learning methods (A^on ^and A^F2F^) performed better on the assessment (p<0.001). There was no difference between online and F2F settings (p=0.325).

**Table 2:** Mean values (±SD) of the percentage correct answers on ETW diagnosis evaluation (25 intraoral photographs) for online and F2F settings ^A, B^No significant difference between online and F2F strategy (p=0.583). ^a, b^Significant difference between lecture and lecture+active learning groups (p<0.01) (two-way ANOVA). ANOVA: analysis of variance; ETW: erosive tooth wear; F2F: face-to-face

Group	^On ^(Online)		^F2F ^(Face-to-face)	
	n	Quiz	n	Quiz
L (lecture)	8	47.0 (±6.68)^Aa^	12	47.8 (±15.7)^Aa^
A (lecture+active learning)	8	69.5 (±12.5)^Ab^	16	64.7 (±10.5)^Ab^

In the online setting, the addition of active learning (A) compared to lecture-only (L) resulted in a very large effect (d=2.25), indicating a substantial improvement in diagnostic ability. In the F2F setting, the effect was also large (d=1.26), suggesting meaningful gains in practical performance. When comparing settings (online vs. F2F), the effect sizes were negligible for the lecture groups (d=0.07) and small for the active learning groups (d=0.42), confirming the absence of significant differences between environments. These findings indicate that active learning enhanced diagnostic ability regardless of setting, with similar effectiveness in both online and F2F formats.

**Figure 2:**
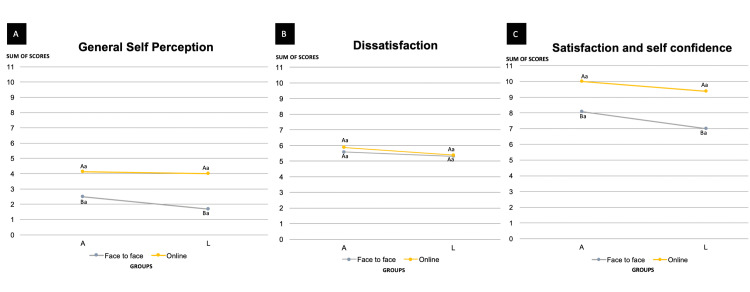
Self-perception of the students with the suggested activities A. Mean of general self-perception answers (dissatisfaction, satisfaction, and self-confidence questions): Different uppercase letters indicate a significant difference between the sum of satisfaction online (On) and F2F setting (p=0.001), whereas equal lowercase letters indicate no significant difference between lecture and active learning groups (p=0.405). No significant interaction was found between setting and group (p=0.540) (two-way ANOVA and Tukey’s test). Standard deviation: A^F2F^=1.57, A^on^=1.25, L^F2F^=2.06, L^on^=1.85. B. Mean of dissatisfaction: Equal uppercase letters indicate no significant difference between dissatisfaction of online and F2F setting (p=0.394), and equal lowercase letters indicate no significant difference between lecture and active learning groups (p=0.068). No significant interaction was found between setting and group (p=0.581) (two-way ANOVA and Tukey’s test). Standard deviation: A^F2F^=0.515, A^on^= 0.354, L^F2F^=0.873, L^on^=0.518. C. Mean of satisfaction and self-confidence: Different uppercase letters indicate a significant difference between satisfaction and self-confidence of online and F2F settings (p<0.001), and equal lowercase letters indicate no significant difference between lecture and active learning groups (p=0.082). No significant interaction was found between setting and group (p=0.635) (two-way ANOVA and Tukey’s test). Standard deviation: A^F2F^=1.44, A^on^=1.07, L^F2F^=1.71, L^on^=1.60. F2F: face-to-face; L^F2F^: lecture face-to-face; A^F2F^: active face-to-face; L^On^: lecture online; A^On^: active online

Figure 2 illustrates the students' self-perception regarding the activities. The overall analysis of general self-perception (dissatisfaction, satisfaction, and self-confidence together) (Figure 2A) revealed a significant difference between the online and F2F settings (p=0.001), with students in the online group showing higher scores, meaning a positive self-perception of one's own performance during the activity. No significant difference was observed between the L and A groups (p=0.405), nor was there any interaction between setting and group (p=0.540). Regarding dissatisfaction (Figure 2B), no significant differences were found between the online and F2F settings (p=0.394) or between the L and A groups (p=0.068), and no interaction was detected between setting and group (p=0.581). For satisfaction and self-confidence (Figure 2C), a significant difference was observed between the online and F2F settings (p<0.001), indicating that students in the online activity reported higher levels of satisfaction and self-confidence compared to those in the F2F setting, with no effect of group (L or A) (p=0.082) and no interaction between setting and group (p=0.635).

## Discussion

This study aimed to evaluate the impact of active learning on dental students' ability to diagnose ETW and whether the environment in which it was applied influenced performance. In terms of the diagnosis of ETW, active learning improved the diagnostic ability of students, regardless of the environment in which it was implemented, leading to the rejection of hypothesis H1 and the acceptance of hypothesis H2.

According to the European core curriculum in cariology, dental students must be equipped with the competence to use their knowledge of biological, medical, and clinical sciences to identify caries and make informed decisions about prevention and management at both individual and population levels [19]. Similarly, in the context of ETW, it is essential to enable students to detect pathological wear at an early stage, thereby promoting timely and appropriate interventions [9]. As observed in the present study, in terms of the clinical diagnostic performance of ETW, participation in active learner-centered activities is essential, as these activities led to significantly better outcomes in both online and F2F settings. This trend has also been observed in other studies [9,18,20-22]. Active learning strategies have been shown to improve engagement, increase attention, and enhance student performance [23-25]. This study presents some limitations that should be acknowledged when interpreting the results. These methods also support the development of lifelong learning skills and are considered more time-efficient than traditional lectures. Therefore, active learning, even in online settings, appears to be a promising approach to enhance skills and reduce ETW diagnostic errors by promoting a deeper understanding of the subject matter. Moreover, optimizing ETW diagnosis through online active learning methods is particularly valuable, as it addresses the challenges of remote education while enabling broader implementation across educational institutions. This can help expand the knowledge base on the diagnosis of ETW and improve consistency in clinical training.

The analysis of effect sizes provided further insight into the practical relevance of the findings. For diagnostic ability, the active learning strategy demonstrated a very large effect in the online environment (d=2.25) and a large effect in the F2F setting (d=1.26), confirming its strong impact on students’ clinical diagnostic performance. These results emphasize that active learning substantially enhances students’ practical skills, particularly in online settings, where interactive and guided participation may compensate for the physical absence of the tutor. Conversely, the comparison between learning environments revealed negligible to small effect sizes (d=0.07 for L and d=0.42 for active learning), indicating that the performance of students was comparable between online and F2F modalities. This suggests that, when properly structured, online active learning can achieve learning outcomes equivalent to those of traditional classroom settings. Taken together, these findings support the integration of active learning methods in hybrid dental education and highlight their potential to strengthen clinical competence, even in virtual contexts.

When students' performance in theoretical knowledge acquisition was analysed, lectures alone did not result in significant improvement in the online group. Unlike typical online courses in which students participate from home, often facing distractions and the risk of procrastination, the intervention was conducted in a single supervised space with computers or tablets and headphones, helping to maintain student engagement. This controlled setting may have mitigated some common challenges associated with online learning and could limit the generalizability of the findings. Research conducted by Al-Natour et al. revealed that, while students appreciated the flexibility of online courses, many struggled to maintain focus during sessions, highlighting the need for interactive and engaging methods to sustain attention [26]. Similarly, a study conducted by Li et al. compared the problem-based learning (PBL) approach in F2F and online learning settings [15]. Although the overall performance was better among students who participated in the F2F activity, students in the distance learning group reported improvements in communication skills, decision-making abilities, self-directed learning, and peer-to-peer interaction. These findings support the idea that interactive learning strategies can enhance engagement and knowledge acquisition in virtual environments. Thus, one possible explanation for this result may be the sense of disconnection, which may impact engagement to obtain knowledge. Interestingly, when the active learner-centered activity was added, it led to enhanced understanding of ETW theoretical knowledge. This finding is noteworthy since the active learning strategy was originally designed for clinical diagnosis. However, it appears that the opportunity to ask questions and clarify information during tutoring sessions may have reinforced students' theoretical knowledge. Additionally, tutored guided session seems to provide a structured and interactive learning experience, potentially increasing student motivation. This reinforces the idea that interactive elements play a crucial role in knowledge retention, particularly in online settings where engagement levels might otherwise be lower.

For the F2F strategy, lecture-based learning alone was sufficient to improve students' theoretical knowledge about ETW, with no additional improvement when active learner-centered education was incorporated. This suggests that the direct interaction inherent in F2F could be sufficient to sustain student engagement in theoretical classes.

The study also revealed interesting differences in students' self-perception between online and F2F settings. The online group demonstrated significantly higher levels of satisfaction and self-confidence compared to their F2F counterparts. This aligns with Li et al. who reported that students engaged in online PBL activities exhibited a more positive attitude toward the learning process [15]. Although a significant difference was found in satisfaction and self-confidence levels, there were no significant differences in dissatisfaction between the two settings. One explanation for the increased satisfaction and self-confidence in the online group could be the flexibility and convenience associated with online learning. The ability to complete tasks at one’s own place may have contributed to a sense of control, thereby enhancing both satisfaction and self-confidence. Additionally, online formats may reduce the pressure associated with immediate performance and public speaking, which are more common in F2F settings, allowing students to feel more comfortable and confident in their abilities. Another contributing factor could be the reduced risk of judgment from peers and instructors in virtual environments, fostering a safer space for engagement and participation.

These findings regarding satisfaction and self-confidence are particularly relevant given that the dental students who participated in this research are part of Generation Z, individuals born between 1996 and 2012 [27]. This generation is characterized by a strong reliance on technology, a preference for sensory-rich, interactive content, and a desire for frequent, personalized feedback. As López-Santacruz and Guízar-Mendoza emphasize, it is important to incorporate constant technological engagement, effective communication strategies, and educational approaches that promote creativity, well-being, and social responsibility [28]. Furthermore, for this generation, prioritizing the development of practical skills alongside theoretical knowledge is essential [28]. Research by Buajeeb et al. also supports this, showing that interactive tools, such as educational games, enhance both engagement and knowledge retention, demonstrating the effectiveness of online and hybrid learning models [29]. In this way, interactive online discussions provide a valuable platform for two-way communication, allowing students to engage in real-time participation, pose questions, and build meaningful connections with instructors and peers, as demonstrated by active learning strategies in the present study.

The study sample consisted of a convenience cohort of third-year dental students, which may limit the generalizability of the findings. However, this group represents the target population for which the educational intervention was designed, providing relevant insights into the effectiveness of the hybrid learning model in undergraduate dental education. Although the sample size was relatively small, the results demonstrated statistically significant differences between groups, indicating that the study had sufficient power to detect meaningful effects. Furthermore, the groups were homogenous in their residual theoretical knowledge of ETW, as shown by preliminary assessments that revealed comparable knowledge levels across both online and F2F groups. This homogeneity supports the validity of the findings. 

In the present study, the online component of the hybrid learning model was conducted in a controlled environment to ensure that all participants were actively engaged and had access to the training. In typical remote learning scenarios, it is not possible to verify whether students are truly attending or paying attention to the content, which can affect learning outcomes. Therefore, the findings should be interpreted with the understanding that they reflect conditions in which students are present and actively participating. This methodological choice strengthens internal validity but may limit the generalizability of the findings to broader, uncontrolled online learning contexts. Future research should examine how hybrid learning performs in more authentic educational environments, where variability in engagement and access more closely reflects real-world conditions.

## Conclusions

Based on the results, it can be concluded that when suitable strategies are implemented, knowledge acquisition in ETW, both in terms of theoretical understanding and diagnostic performance, can become comparable between online and F2F settings. As dental education undergoes this transformation, continuous research and assessment are essential to refine online learning environments, ensuring that students receive high-quality educational experiences and are well-prepared for the dynamic challenges of modern dentistry.

## Appendices

Knowledge questionnaire about erosive tooth wear (ETW)

Group: ________   

1.I believe the surface most affected by erosive tooth wear is: ( ) Buccal        ( ) Lingual/Palatal ( ) Proximal    (x) Occlusal

2.I believe the tooth most affected by erosive tooth wear is: ( ) Incisor   ( ) Premolar (x) Molar    ( ) All teeth are equally affected

3.I believe that dental erosion can be caused: ( ) By excessive sugar consumption (x) By extrinsic and intrinsic acids ( ) By an aggression during tooth formation ( ) By mechanical aggression on the tooth

4.Erosive tooth wear is: ( ) A subsurface lesion (x) The result of a chemical-mechanical process ( ) A reversible lesion ( ) All of the above

5.The early clinical sign of initial erosive tooth wear is: ( ) Shortening of the teeth ( ) Facial muscle tension (x) Smoothing of enamel surfaces ( ) Elevation of restorations

6.To diagnose erosive tooth wear: ( ) Clinical and radiographic examination are required ( ) Clinical exam and anamnesis about sugar consumption frequency (x) Clinical exam and anamnesis about eating habits, brushing habits, and medical history ( ) Clinical exam, radiographs, and anamnesis about eating habits

7.To prevent dental erosion, patients should be advised to: (x) Reduce acidic beverage consumption ( ) Reduce sugar consumption ( ) Increase brushing frequency ( ) Take medications that reduce salivary flow

8.Erosive tooth wear is considered pathological: ( ) When more than 50% of the surface is worn ( ) When the tooth shows loss in height (x) When the worn tooth will not survive functionally throughout the individual’s lifespan ( ) All of the above

9.Which form of vitamin C ingestion minimizes erosive tooth wear: (x) Tablet swallowed at once ( ) Effervescent tablet ( ) Chewable tablet ( ) Liquid form

10.What is the recommended waiting time after consuming acidic foods before brushing teeth? ( ) Immediately    ( ) 30 minutes ( ) 1 hour              (x) Any interval

11.Which treatment is recommended for maxillary incisors with BEWE score 3 and esthetic compromise: ( ) Act on causal factors ( ) Act on causal factors and perform porcelain veneers ( ) Act on causal factors and apply sealants (x) Act on causal factors and restore with composite resin

12. Based on the table below showing a patient’s dietary diary, classify the risk of erosive tooth wear:

**Table 3:** Patient dietary diary with corresponding classification of ETW risk ETW: erosive tooth wear

Dietary Diary	Saturday	Sunday	Monday	Tuesday
7 AM	Kiwi and orange with milk+bread	Coffee with milk and bread	Milk with strawberry and cheese	Banana with cereal
10 AM	3 filled cookies	1 chocolate bar	1 banana	5 nuts
1 PM	Rice, beans, meat+cola	Pasta and beer	Salad with vinegar and pastry	Rice, beans, mayonnaise+beer
4 PM	Coffee with cake	Sweet popcorn	Strawberry yogurt with granola	Apple
7 PM	Bread, meat, cheese+orange juice	Salad without dressing and milk	Bread with cheese and tomato+wine	Chocolate crepe+wine
10 PM	Cheese and wine	Lemon yogurt	Milk with chocolate	Cheese with guava paste
